# Yeast Methylotrophy and Autophagy in a Methanol-Oscillating Environment on Growing *Arabidopsis thaliana* Leaves

**DOI:** 10.1371/journal.pone.0025257

**Published:** 2011-09-26

**Authors:** Kosuke Kawaguchi, Hiroya Yurimoto, Masahide Oku, Yasuyoshi Sakai

**Affiliations:** 1 Division of Applied Life Sciences, Graduate School of Agriculture, Kyoto University, Kyoto, Japan; 2 Research Unit for Physiological Chemistry, The Center for the Promotion of Interdisciplinary Education and Research, Kyoto University, Kyoto, Japan; 3 CREST, Japan Science and Technology Agency, Tokyo, Japan; Institute of Developmental Biology and Cancer Research, France

## Abstract

The yeast *Candida boidinii* capable of growth on methanol proliferates and survives on the leaves of *Arabidopsis thaliana*. The local methanol concentration at the phyllosphere of growing *A. thaliana* exhibited daily periodicity, and yeast cells responded by altering both the expression of methanol-inducible genes and peroxisome proliferation. Even under these dynamically changing environmental conditions, yeast cells proliferated 3 to 4 times in 11 days. Among the C1-metabolic enzymes, enzymes in the methanol assimilation pathway, but not formaldehyde dissimilation or anti-oxidizing enzymes, were necessary for yeast proliferation at the phyllosphere. Furthermore, both peroxisome assembly and pexophagy, a selective autophagy pathway that degrades peroxisomes, were necessary for phyllospheric proliferation. Thus, the present study sheds light on the life cycle and physiology of yeast in the natural environment at both the molecular and cellular levels.

## Introduction

In nature, microbe–plant interactions are a critical part of carbon circulation. After plants die, microorganisms living on plant surfaces and in the soil decompose plant materials into small compounds that can be reused as nutrients by the next generation and other organisms. Since non-phytopathogenic microorganisms on plant surfaces cannot invade the plant to obtain nutrients, they must survive on plant surfaces, even while the plants are living.

Methanol is an intermediate of the global methane cycle, which is the carbon circulation cycle between the two major green house gases methane and CO_2_. It is estimated that approximately 1 Gt of methane per year diffuses into the oxic environment and is oxidized by aerobic microbes to CO_2_ (0.6 Gt per year) via methanol [1]. In addition, a large amount of methanol is thought to be present as methylesters in plant cell wall constituents, such as pectin.

Methylotrophic bacteria and yeast are microorganisms that can grow using methanol as a single carbon and energy source. Since the methylotrophic yeast *Candida boidinii* was first isolated in 1969 [2], many methylotrophic yeast strains have been isolated from plant materials [3], [4], e.g., forest soils, fallen leaves, and the skins of olives and grapes [5]. The association and symbiotic relationship between plants and methylotrophic bacteria is well documented [6]–[8], but the interaction between methylotrophic yeasts and plants has not been studied. Additionally, the amount of atmospheric methanol emitted from plant leaves was previously estimated using a gas chamber [9], but these results have not been used to assess the local methanol dynamics in the meso-environment or to determine how microorganisms use methanol in a physiological context. Recently, plant leaves were shown to emit methane [10]. The phyllosphere is thought to contain a higher concentration of aqueous methanol than gaseous methane, and the presence of methanol may affect biological methane consumption since methanol competitively inhibits methane consumption through methane monooxygenase.

Although the ecology of methylotrophic yeasts is still relatively unknown, the biochemistry and cell biology of methylotrophic yeasts have been extensively studied at the molecular level using *C. boidinii*, *Pichia pastoris* and *Hansenula polymorpha*. Methanol is first oxidized by alcohol oxidase (encoded by *AOD1* in *C. boidinii*) to form formaldehyde and H_2_O_2_. In the assimilation pathway, formaldehyde is fixed to xylulose 5-phosphate by dihydroxyacetone synthase (encoded by *DAS1*), forming dihydroxyacetone and glyceraldehyde 3-phosphate, and assimilated for biosynthesis of cell constituents. Alcohol oxidase and dihydroxyacetone synthase are localized to the peroxisome, together with the anti-oxidant enzymes peroxisomal catalase (encoded by *CTA1*) and peroxiredoxin Pmp20 (encoded by *PMP20*). In the dissimilation pathway, formaldehyde is oxidized through the sequential reactions catalyzed by glutathione-dependent formaldehyde dehydrogenase (encoded by *FLD1*), *S*-formylglutathione hydrolase (encoded by *FGH1*), and formate dehydrogenase (encoded by *FDH1*) to yield CO_2_ and NADH. In previous studies with *C. boidinii*, we revealed that knockouts for several methanol-inducible genes, notably the *aod1Δ*, *das1Δ*, *pmp20Δ*, and *fld1Δ* strains, lost their ability to grow on methanol [11]–[15]. In contrast, the *FGH1*, *FDH1*, and *CTA1* genes were dispensable for growth on methanol, although these genes were induced in methanol-grown cells.

Another remarkable feature of yeast methylotrophy is the high up-regulation and down-regulation of methanol-metabolizing enzymes and peroxisomes. When cells are transferred from a glucose to methanol medium, two representative peroxisomal enzymes, alcohol oxidase and dihydroxyacetone synthase, are induced approximately 3000- to 10000-fold. Since methanol-induced peroxisomes are robust and their homeostasis is easily controlled by the carbon source, methylotrophic yeasts have been used as valuable experimental systems to elucidate the molecular mechanisms of peroxisome biogenesis and degradation [16], [17]. Peroxisomes are degraded by pexophagy, a type of autophagy. Studies with methylotrophic yeasts have helped identify peroxine genes (*PEX*) that are involved in peroxisome biogenesis and autophagy genes (*ATG*), both of which are conserved from yeast to mammals. However, it is still unknown why methylotrophic yeasts have giant peroxisomes and how methanol-mediated induction and down-regulation are physiologically significant. In this report, we examined the survival, proliferation, and cellular physiology of the methylotrophic yeast *C. boidinii* on the surfaces of living *Arabidopsis thaliana* leaves and determined the daily oscillation pattern in methanol concentrations at the phyllosphere.

## Results

### Cell-based methanol assay using *C. boidinii* cells expressing Venus-PTS1 under the *DAS1* promoter

**Figure 1:**
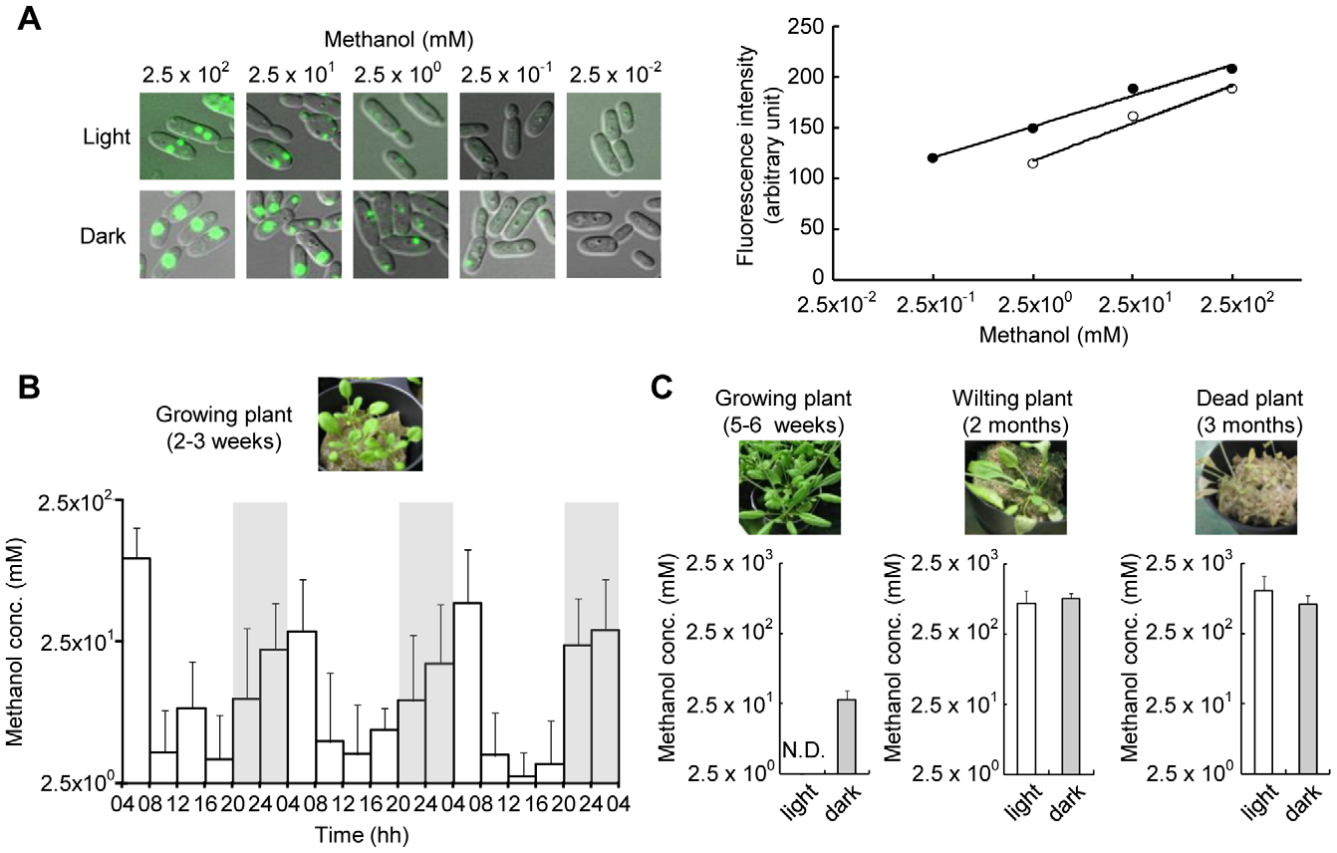
The *C. boidinii* methanol sensor. (A) Standard curve for fluorescent intensities of Venus-PTS1 relative to the methanol concentrations in the agar plates. Symbols indicate the following: white circle, light conditions; black circle, dark conditions. (B) Methanol concentrations on growing *A. thaliana* leaves (2–3 weeks after germination). Leaves were inoculated with the *C. boidinii* PDAS strain at 4, 8, 12, 16, 20, 24 hh, and the fluorescent intensity was measured 4 h post inoculation. The methanol concentration represents the average of at least 50 cells. Error bars show the standard deviations from at least three independent experiments. Gray bars indicate the dark period. (C) Methanol concentrations on *A. thaliana* leaves at various plant ages. Left, growing plant (5–6 weeks after germination); center, wilting plant (2 months after germination); right, dead plant (3 months after germination). Error bars show the standard deviations from at least three independent experiments.

We established an experimental protocol in which a fluorescent protein Venus tagged with peroxisome targeting signal 1 (Venus-PTS1) under a methanol-inducible promoter responded linearly to environmental methanol concentrations (Materials and Methods). For this purpose, we selected the dihydroxyacetone synthase (*DAS1*) promoter because of its strong and specific response to methanol [18]. After the inoculation of the constructed *C. boidinii* PDAS strain, and 4 h in the dark or light, the cellular fluorescence intensity was proportional to the methanol concentration in the agar plates and was within methanol concentrations of 2.5–250 mM under light conditions and 0.25–250 mM under the dark conditions (Figure 1A). This difference of standard curves from cells inoculated under light or dark condition may be due to quenching of fluorescent proteins under light condition. According to the experimental condition used, we estimated the local methanol concentration in the last 4 h based on the fluorescence intensity.

### Methanol dynamics on *A. thaliana* leaves

The local methanol concentration at the phyllosphere of growing *A. thaliana* was determined by inoculating the *C. boidinii* PDAS strain onto the upper side of a young leaf (2–3 weeks after germination). The estimated methanol concentration on the leaves changed during the daily light–dark cycle and was higher in the dark period (20–4 hh, 0.048–0.086%, 14.6–26.2 mM) and morning (4–8 hh, 0.21%, 64 mM) but lower in the light period (8–20 hh, 0.014–0.015%, 4.26–4.57 mM) (Figure 1B). As shown in Figure 1C, this daily oscillation in methanol was also observed at the phyllosphere in adult *A. thaliana* (5–6 weeks after germination). Growing plants kept in the dark lost this oscillation in methanol concentrations and had methanol concentrations higher than 250 mM. Similarly, when the plants aged and wilted or died (>2 months), the methanol concentration exceeded 250 mM and did not oscillate (Figure 1C). These results show that the methanol concentration at the phyllosphere changes dynamically during the daily light–dark cycle and that plant aging or death allows the phyllospheric environment to maintain high methanol concentrations.

The presence of some sugar, which represses the methanol-inducible expression in *C. boidinii* may cause apparent decrease in methanol concentration on plant leaves. To exclude this possibility, we inoculated the sensor cells on plant leaves at 12 hh apparently having low methanol concentration with known concentrations of methanol. We confirmed the expression of Venus-PTS1 fluorescence in sensor cells corresponding to the expected methanol concentration (Figure S1). Therefore, we concluded that there was no sugar on *Arabidopsis* leaves sufficient to repress the expression of methanol-inducible genes. The yeast methanol sensor may respond to pectin methylester at phyllosphere. When the yeast cells were spotted on pectin methylester powder (DE 90%) [19], the fluorescence level corresponded to less than 0.2 mM methanol, and the observed methanol concentration was much higher at the phyllosphere. Therefore, yeast cells responded to free methanol.

### *C. boidinii* proliferation at the phyllosphere of growing *A. thaliana*

**Figure 2:**
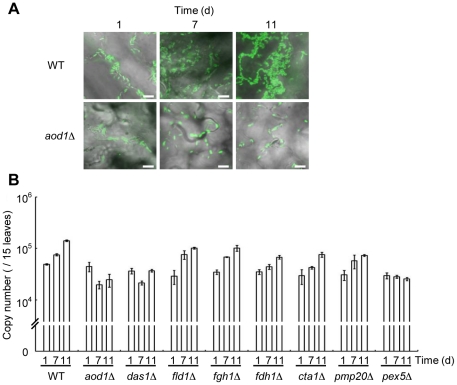
Proliferation of *C. boidinii* on growing *A. thaliana* leaves. (A) Confocal microscope images of the Venus-labeled wild-type strain and *aod1*Δ strain on a plant leaf. *C. boidinii* cells were spotted on *Arabidopsis* leaves (2–3 weeks after germination). Bar, 10 µm. (B) Quantitation of cell number of the wild-type, and knockout strains after 1, 7, and 11 days. Error bars show the standard deviations of triplicate measurements.

Careful observations of *C. boidinii* cells at the phyllosphere indicated that the number of *C. boidinii* cells increased at the leaf surface of growing *A. thaliana* (Figure 2A). In addition, a quantitative PCR analysis (qPCR) was conducted over approximately 2 weeks by inoculating the leaves of growing *A. thaliana* (2–6 weeks after germination), which exhibited oscillations in methanol concentrations, with yeast cells. Both the fluorescence images and qPCR analyses showed that *C. boidinii* cells could proliferate approximately 3–4 times after 11 days of inoculation (Figure 2A and B). Similarly, *P. pastoris* cells also proliferated at the phyllosphere (Figure S2).

### Induction of methanol metabolism on the plant leaf surface

**Figure 3:**
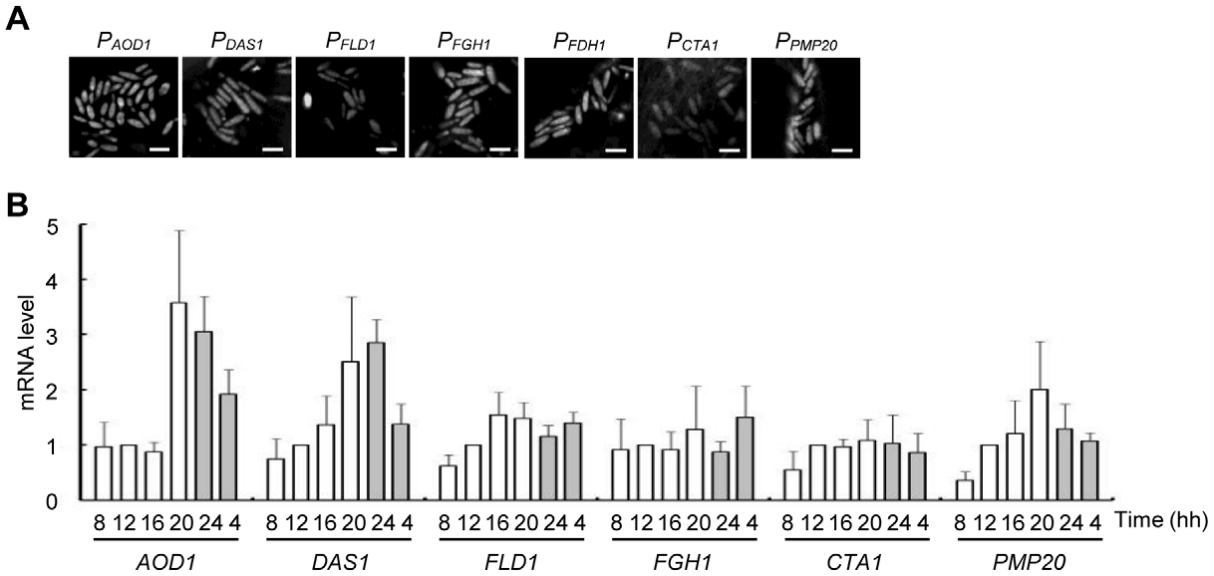
Expression of methanol-inducible genes on growing *A. thaliana* leaves. Methanol-inducible genes were expressed on *A. thaliana* leaves and their expression levels oscillated during the daily light–dark cycle. (A) Confocal microscope images of *C. boidinii* that was inoculated on the plant leaf surface. Venus was expressed under the control of the following promoters: A, *AOD1* B, *DAS1* C, *FGH1* D, *FLD1* E, *FDH1* F, *CTA1* G, *PMP20*. Bar, 5 µm. (B) mRNA levels of methanol-inducible genes during the daily light–dark cycle. Expression level is expressed as the relative value to the sample collected at 12 hh. Error bars show the standard deviations of triplicate measurements.

To test whether methanol-inducible genes other than *DAS1* were also induced on the plant leaf surface, we constructed various *C. boidinii* cells expressing Venus under the control of the methanol-inducible promoters *AOD1*, *FLD1*, *FGH1*, *FDH1*, *CTA1*, and *PMP20*. Cytosolic fluorescence was only detected in the strains that were incubated on synthetic methanol (SM) agar plates, and not synthetic glucose (SD) agar plates. Next, we inoculated growing *A. thaliana* leaves with these strains. As shown in Figure 3A, cytosolic fluorescence was detected with all of the tested strains, especially during the dark period.

We further quantitated the transcripts of these methanol-metabolizing genes. Total RNA was extracted from proliferating *C. boidinii* at the phyllosphere and subjected to qRT-PCR analysis, as described in the Materials and Methods. The transcriptional levels of the peroxisomal genes *AOD1*, *DAS1*, *CTA1*, and *PMP20* peaked at 20 hh or 24 hh when the methanol concentration was relatively high (approximately 25 mM). In contrast, the transcript levels of these peroxisomal genes were the lowest at 8 hh in the light period (Figure 3B). The expression pattern of the cytosolic formaldehyde dehydrogenase gene (*FLD1*) and *S*-formylglutathione hydrolase gene (*FGH1*) also exhibited a similar pattern, although the induction in the dark period was not so apparent. In general, the expression of methanol-inducible genes was the highest in the dark period (20 hh or 24 hh) and lowest in the light period (8 hh). Furthermore, the expression of these methanol-inducible genes peaked before the maximum methanol concentration was reached.

### Assimilation of methanol is required for yeast proliferation at the plant leaf surface

We wanted to determine whether the methanol-inducible genes involved in yeast methanol metabolism are necessary for proliferation on *Arabidopsis* leaves. We examined the proliferation of each knockout and wild-type strain on a plant leaf by fluorescent microscopy and qPCR analyses. In the wild-type strain, both the number of fluorescent cells and the Venus gene copy number increased after 11 days of incubation (Figure 2A and B). On the other hand, neither increased with the *aod1*Δ nor *das1Δ* strains where the Venus gene copy number was one-tenth of the wild-type strain (Figure 2B). These results indicate that *C. boidinii* proliferation at the phyllosphere was supported by methanol assimilation at the plant leaf surface.

We previously showed that *C. boidinii* can grow on pectin [19]. During growth on pectin, pectin is hydrolyzed to methanol and polygalacturonate by the yeast pectin methyl esterase. While knockout strains of methanol-metabolizing enzymes exhibited a severe defect in growth on methanol, all of these knockout strains retained the ability to grow on pectin. Since both *aod1Δ* and *das1Δ* could not grow on plant leaves, free methanol was determined to be the main carbon source for assimilation on growing *Arabidopsis* leaves, and the methanol moiety of pectin from the plant cell wall was not utilized efficiently. Unexpectedly, mutant strains impaired in either the formaldehyde dissimilation pathway (*fld1Δ* and *fdh1Δ* strains) or peroxisomal anti-oxidant enzymes (catalase and Pmp20) did not show significant impairment in proliferation on growing plant leaves. These findings indicate that yeast metabolism at the phyllosphere is distinct from the metabolism that occurs during growth on methanol under laboratory conditions.

### Yeast peroxisome dynamics on plant leaves

The expression of the peroxisomal genes *AOD1*, *DAS1*, and *PMP20* corresponded with the daily light–dark cycle, which prompted us to examine peroxisome dynamics on growing leaves using a *C. boidinii* strain expressing Venus-PTS1 under the constitutive *ACT1* promoter.

**Figure 4:**
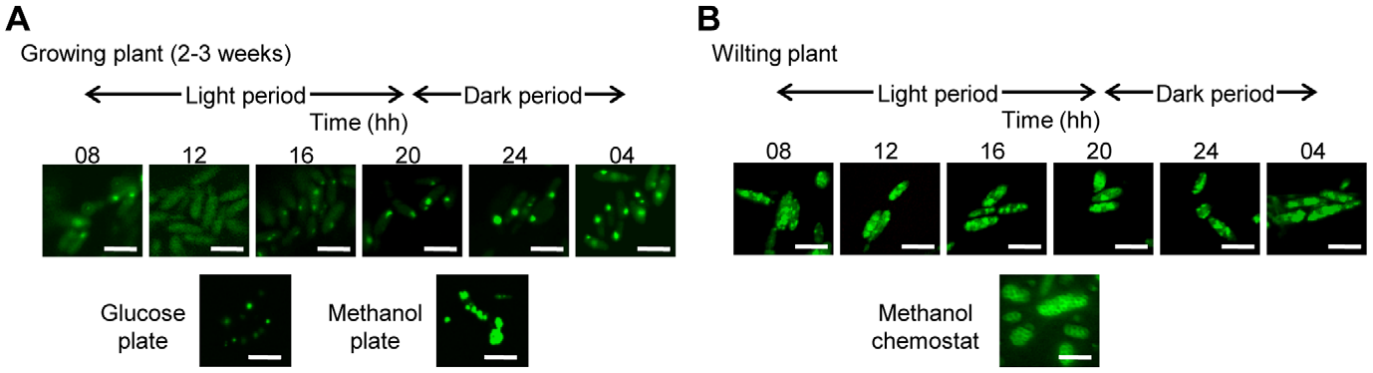
Peroxisome dynamics on growing and wilting plant leaves. Peroxisomes were observed in *C. boidinii* expressing Venus-PTS1 under the control of the *ACT1* promoter. *C. boidinii* cells inoculated on (A) (upper) a growing plant leaf, (lower) synthetic glucose and methanol plates, (B) (upper) wilting plant leaf observed at the indicated times, and (lower) methanol-limited chemostat culture at a dilution rate of 0.05 h^−1^. Bar, 5 µm.

Cells inoculated on a SD plate had one or two small peroxisomes. Cells growing on SM agar plates exhibited fluorescence with large and clustered peroxisomes (Figure 4) [20]. Cells on growing *Arabidopsis* leaves contained one or two peroxisomal dots, similar to glucose-grown cells, with some cytosolic fluorescence at 8 hh (Figure 4A). In contrast, at 24 hh in the dark period, peroxisomal fluorescence increased with the simultaneous disappearance of cytosolic fluorescence. These results suggest that peroxisomes are slightly induced without a significant increase in the number of peroxisomes and that peroxisomal transport efficiency increases at 24 hh in the dark period (Figure 4A). In addition, these findings indicate that peroxisome biogenesis in *C. boidinii* responds to the environmental conditions on the plant leaf. Peroxisomal dot fluorescences could not be observed in the *pex5Δ* strain expressing Venus-PTS1 under the control of *ACT1* promoter on the plant leaves (Figure S3). Furthermore, the *pex5Δ* strain did not proliferate on growing plants (Figure 2B). Therefore, *C. boidinii* proliferation on growing plant leaves requires proper peroxisome assembly.

Giant peroxisomal fluorescence, similar to those observed in methanol-limited chemostat cultures at a low dilution rate [21], was observed in *C. boidinii* on the leaves of wilting or dead plants (Figure 4B). Since *DAS1* was highly induced under these conditions, dihydroxyacetone synthase (possibly together with alcohol oxidase) is abundant in these cells. However, the wild-type and *aod1Δ* strains of *C. boidinii* did not proliferate on these aging plants (Figure S4). We conclude that peroxisomes function as storage organelles as further outlined in the Discussion.

### Importance of yeast autophagy and pexophagy in yeast proliferation at the phyllosphere

Autophagy is a degradation pathway for cytosolic components, including organelles, that occurs after these components are transported into the vacuole [22]. Autophagy is thought to recycle amino acids or remove nonessential organelles and proteins. Conventional *ATG* genes are required for all autophagic pathways [23]. In addition to these conventional *ATG* genes, pexophagy, i.e., the autophagic pathway that specifically degrades peroxisomes, requires the pexophagy-specific gene *ATG30* [24]. However, yeast *atg* mutants do not exhibit clear growth phenotypes, and thus the physiological significance of autophagy in yeast is still unknown.

To determine the physiological significance of autophagy and pexophagy during yeast proliferation at the phyllosphere, we cloned *CbATG1* (a pivotal kinase for all autophagic pathways), *CbATG8* (a marker of autophagic membranes) [23], and *CbATG30*, and derived the *Cbatg1Δ*, *Cbatg8Δ*, and *Cbatg30Δ* strains, which had lost autophagic and/or pexophagic activity (Figure S5).

**Figure 5:**
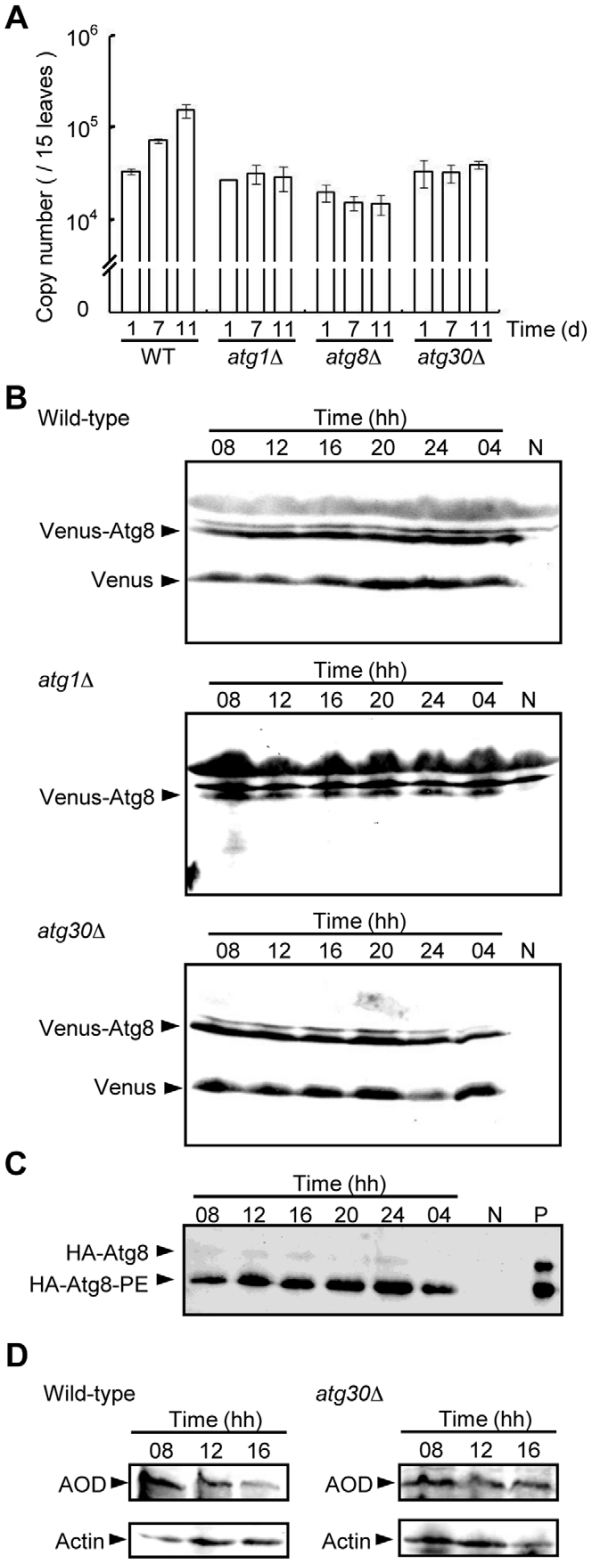
Autophagy and pexophagy mutants have impaired proliferation on growing *A. thaliana* leaves. (A) Quantitation of the cell mass of the wild-type, *atg1*Δ, *atg8*Δ and *atg30*Δ strains at 1, 7, and 11 days post inoculation. Error bars show the standard deviation of triplicate measurements. (B) Immunoblot analysis of autophagy based on Venus-Atg8 cleavage in the wild-type and *atg1*Δ strains. Cell lysates were prepared from leaves collected at the indicated time points (lanes 1–6 from the left), together with the negative control (lane 7) prepared from uninfected leaves. Arrowheads indicate the band positions corresponding to Venus-Atg8 and the processed Venus. (C) Lipidation of HA-Atg8 in cells of the wild-type strain on growing *A. thaliana* leaves. Yeast cell lysates were prepared from leaves at the indicated time points and was subjected to SDS-PAGE containing 6 M urea. The HA-Atg8 protein was detected by immunoblot analysis. (D) Alcohol oxidase levels in cells of the wild-type and *atg30*Δ strains. Cell lysates were prepared from leaves collected at the indicated time points. Alcohol oxidase and actin were detected by immunoblot analysis as described in Materials and Methods.

To test whether autophagy and pexophagy contribute to yeast proliferation at the phyllosphere, we compared the growth of the *atg* mutant strains with the wild-type strain. The copy numbers of Venus genes in the inoculums for the *Cbatg1*Δ, *Cbatg8Δ*, or *Cbatg30Δ* strains did not increase after 11 days of inoculation (Figure 5A). This indicates that autophagy, including pexophagy, is necessary for yeast proliferation and survival at the phyllosphere.

When Venus-Atg8 is transported into the vacuole via autophagy and exposed to vacuolar proteinases, it is rapidly digested, leaving a free form of Venus that is structurally more resistant to proteinases [25]. Therefore, autophagic activity could be biochemically assessed in Venus-Atg8–expressing cells, and the *Cbatg1Δ* strain, but not the *Cbatg30Δ* strain, was shown to have impaired starvation-induced autophagy (Figure S6). During autophagy and autophagosome formation, Atg8 is lipidated by a ubiquitin-like conjugation system [26]. The lipidated and non-lipidated forms of CbAtg8 can be distinguished using an HA-tagged CbAtg8 and urea-SDS-PAGE as previously described [27], [28]. When pexophagy was induced by shifting methanol-grown cells to an ethanol medium, peroxisomal alcohol oxidase degradation via pexophagy was blocked not only in the *Cbatg1Δ* and *Cbatg8Δ* strains, but also in the *Cbatg30Δ* strain (Figure S7).

We inoculated growing plant leaves with the Venus-Atg8 and HA-Atg8 strains on a wild-type or *atg1Δ* background and followed autophagy biochemically. We extracted proteins from the Venus-Atg8–expressing cells on plant leaf surfaces and examined the processing of Venus-Atg8. We detected the free Venus form by immunoblot analysis in the wild-type strain that was inoculated on the plant leaf (Figure 5B). In contrast, we could not detect the cleaved Venus form in the *atg1Δ* strain. These results indicated that autophagy is induced throughout the daily dark–light cycle in *C. boidinii* cells on growing plant leaves. This finding is also supported by the observation that the lipidated form of HA-Atg8 is observed throughout the daily cycle (Figure 5C).

We detected cleaved Venus form in the pexophagy-deficient *atg30Δ* strain on growing plant leaves (Figure 5B) indicating that conventional autophagy occurred throughout the daily cycle. On the other hand, from fluorescent peroxisome dynamics analysis, we speculated that pexophagy was induced around 08 hh–12hh. At this time period, we compared alcohol oxidase degradation between wild-type cells and *atg30Δ* cells (Figure 5D). While alcohol oxidase level decreased in the wild-type cells, degradation of alcohol oxidase was not observed in the *atg30Δ* strain. These results showed that conventional autophagy and pexophagy simultaneously occurred at this time period on the plant leaf and were necessary for *C. boidinii* to proliferate at the phyllosphere.

## Discussion

We assessed the dynamics of methanol at the phyllosphere of *A. thaliana* by developing a *C. boidinii* cell sensor. Our analysis determined the local methanol concentration and identified the methanol concentration that elicits responses in yeast cells, which cannot be determined based on the atmospheric methanol concentration in a gas chamber analysis. Based on our analyses, the concentration of free methanol on the leaf surfaces of living plants is 25 mM and is often greater than 250 mM in wilting and dead plants. These unexpectedly high concentrations on the plant surface were a source of methanol. We also observed that methanol was not limited to the area around the stomata and that high concentrations of free methanol were present at all areas of the leaf surface [29]. Therefore, methylotrophs use free methanol at the plant surface. An additional point of interest is the dynamic oscillation of the local methanol concentration at the phyllosphere of growing plants that corresponds to the light–dark cycle. Pectin methylester in the cell wall is thought to be hydrolyzed by the plant pectin methylesterase during cell wall expansion in a regulated manner [30], [31]. However, the source of free methanol and the mechanism and physiology of methanol oscillations at the plant leaf surface, such as the circadian rhythm, are still unknown.

We herein show that methylotrophic yeasts, including both *C. boidinii* and *P. pastoris*, proliferate on the phyllosphere of growing *A. thaliana* where the methanol concentrations oscillated with the daily light–dark cycle. Simultaneously, we established both fluorescent and biochemical procedures that measure cell proliferation, gene expression, and intracellular organelle dynamics. Based on extensive biochemistry, molecular, cell biology, and the molecular breeding system of methylotrophic yeasts, we propose that this methylotrophic yeast–*Arabidopsis* system can be used to explore the yeast life cycle and physiology at the molecular level in nature. Although a gene disruption library has been constructed using the yeast *Saccharomyces cerevisiae*, many yeast mutants have not exhibited a growth phenotype under laboratory conditions, as was the case with autophagy-deficient *atg* mutants. Therefore, the physiological significance and function of many yeast genes are still unknown, especially under natural conditions. The methylotrophic yeast–plant system can combine molecular cellular biology with ecology to create a new platform that will reveal the physiological function of microbes in nature to establish eco-molecular microbiology.

For methylotrophic bacteria, e.g., *Methylobacterium* sp., methylotrophy was shown to support, but not to be essential, for growth at the phyllosphere [32]. In this study, we revealed the common and distinctive metabolic features of methylotrophic growth and phyllospheric proliferation for *C. boidinii*. Methanol assimilation (*AOD1* and *DAS1*) and peroxisome assembly (*PEX5*) were both required for *C. boidinii* to grow using methanol as a single carbon and energy source and were strictly required for phyllospheric growth on growing plants. These findings indicated that methanol is the only carbon source for *C. boidinii* assimilation at the phyllosphere. Another interesting finding is that autophagy and pexophagy are required for yeast proliferation at the phyllosphere (see below for further discussion).

Regarding the methanol dissimilation pathway, the *fld1Δ* strain, but not the *fdh1Δ* or *fgh1Δ* strains, completely lost the ability to grow on methanol as a carbon source [13], [37], [38]. This difference in growth is because the *fgh1Δ* and *fdh1Δ* strains can produce one mole NADH/one mole oxidized formaldehyde, while the *fld1Δ* strain cannot produce any NADH through formaldehyde oxidation during growth with methanol as the single energy source. Despite defects in methylotrophic growth, the proliferation of the *fld1Δ* and wild-type strains at the phyllosphere was indistinguishable. Therefore, *C. boidinii* is assumed to acquire energy from other nutrient sources that do not repress the induction of methanol-assimilating enzymes (Figure 2B). The energy and nitrogen sources for *C. boidinii* at the phyllosphere are still unknown. On the other hand, microorganisms at the phyllosphere are thought to suffer from oxidative stresses. Yeast peroxisomes contain two methanol-inducible anti-oxidant enzymes, peroxisomal catalase (*CTA1*) and peroxiredoxin (*PMP20*), and only the latter was required for growth on methanol [11], [12]. Depleting each of these two enzymes did not affect proliferation at the phyllosphere on growing plant leaves, suggesting that some other anti-oxidant system detoxifies the reactive oxygen species that are generated at the phyllosphere.

One interesting finding of this study was the daily and dynamic oscillation of methanol at the phyllosphere of growing plants. Furthermore, yeast cells responded to the daily light–dark cycle and proliferated 3–4 times in 2 weeks, in which C1-metabolism is switched on and off at the transcriptional level. The induction and down-regulation of methylotrophy that occurs in yeast have also been observed in bacteria, and this process may have originated from methanol oscillations at the phyllosphere during the evolution of plant–microbe interactions.

We previously observed huge peroxisomes in *C. boidinii* in methanol-limited chemostat cultures, which reached approximately 80% of the total intracellular volume and contained catalytically non-functional alcohol oxidase crystalline. Similar peroxisomes were observed at the phyllosphere of wilted or dead plants, but yeast cells did not proliferate under these conditions. We think that huge methanol-induced peroxisomes are a storage organelle for proteins as a source of amino acids in the natural environment because motile yeast cells on dead plants must survive until they obtain nutrients for further proliferation.

Gene expression and peroxisome homeostasis seem to occur according to oscillations in the methanol concentration on growing plant leaves. It is noteworthy that the *Cbatg30Δ* strain that has impaired pexophagy (but not impaired in general autophagic pathways) [24] lost the ability to proliferate on plant leaves. These findings indicate that daily organelle turnover plays a critical role in the environmental adaptation and proliferation of *C. boidinii* at the phyllosphere. While autophagy was shown to occur throughout the day, it is still unknown how autophagic activity and selectivity are regulated throughout the daily light–dark cycle.

In previous reports, we and others have shown that autophagy and/or pexophagy in phytopathogenic fungi were involved in the formation of appresoria, the infection process [33]–[35]. However, it is still unknown whether autophagy plays a specific role in the differentiation process or whether autophagy is only necessary for intracellular nutrient recycling. Distinct from these phytopathogens, the present study indicated that pexophagy, which is a process that recovers amino acids and nutrients from peroxisomes, was required for proliferation of methylotrophic yeasts at the phyllosphere.

Environmental methanol dynamics were estimated using a conventional analysis, which is thought to yield the total amount of emitted methanol from metabolism by both plants and microbes. Therefore, the contribution of microbes to the methanol dynamics as well as the related microbe-plant interactions have not been sufficiently examined. In this study, we revealed the life style of methylotrophic yeast at the phyllosphere, i.e., how methylotrophs respond to daily changes in local methanol concentrations using a newly developed yeast methanol sensor. Both plant and microbial C1-metabolic activity could simultaneously and significantly affect the methanol concentration at the phyllosphere and the methane cycle. We think that exploring the physiology of methylotrophs at the phyllosphere, including their responses to and metabolic changes during the daily light–dark cycle, will reveal the mechanisms of the biological methane cycle at the molecular level.

## Materials and Methods

### Yeast strains, and DNA protocols

*C. boidinii* strain TK62 (*ura3*) [36] was used as the wild-type host strain after the indicated expression plasmid was introduced with the *URA3* marker. *C. boidinii* gene-disrupted strains, *aod1*Δ *ura3* [14], *das1*Δ *ura3* [15], *fgh1*Δ *ura3* [37], *fld1*Δ *ura3* [13], *fdh1*Δ *ura3* [38], *cta1*Δ *ura3* [11], *pmp20*Δ *ura3* [12], and *pex5*Δ *ura3* [39], were also used as host strains to express the fluorescent protein derivatives. General DNA protocols, molecular breeding of *C. boidinii*, and primer sequences are described in the supplementary information (See Text S1 and Table S1).

### Plant cultivation and yeast inoculum

*A. thaliana* seeds (Col.) were sown on rock fiber blocks immersed in Hoagland's medium [40] at 4°C in the dark for 2 days, and then grown in a growth chamber at 25°C with 60% humidity and 16 h of illumination per day. To follow yeast proliferation on *A. thaliana*, 1 µl of yeast suspension (OD_600_ = 0.05) was spotted onto the upper side of the leaf. To determine the local methanol concentration on *A. thaliana* leaves, 5 µl of yeast suspension (OD_600_ = 0.5) was spotted onto the upper side of the leaf. A yeast suspension (OD_600_ = 0.05) was sprayed on *A. thaliana* leaves and incubated for 5 days to prepare the samples for qRT-PCR and immunoblot analysis.

### Quantitation of yeast cell numbers on *A. thaliana*

After inoculating *A. thaliana* leaves with *C. boidinii*, 15 leaves were collected to quantify the number of yeast cells. The leaves were stored at −80°C until further analysis. To isolate genomic DNA, the leaves were suspended in SCEM buffer (0.1 M Tris-HCl pH 7.5, 0.1 M EDTA, 0.9 M sorbitol, and 30 mM β-mercaptoethanol) containing 0.1 mg/ml Zymolyase 100T and incubated at 37°C for 1 h. Next, 1% SDS was added and the samples were incubated at 65°C for 30 min. Then, an equal amount of phenol/chloroform mixture was added and the samples were vortexed and centrifuged. The supernatants were collected into new tubes and the DNA was precipitated with 2-propanol. The DNA was resuspended in TE buffer (10 mM Tris-HCl pH 7.5 and 1 mM EDTA) and treated with RNase. Then, the DNA was precipitated with ethanol and resuspended in nuclease-free water, and subjected to qPCR analysis.

### Preparation of cell extracts from yeast cells on plant leaves

Leaves were suspended in lysate buffer (0.25 N NaOH, 150 mM β-mercaptoethanol, and 0.1% Triton X-100) and incubated at 4°C for 10 min. To examine Atg8-lipidation, cell lysates were prepared by suspending the leaves in 50 mM potassium phosphate buffer (pH 7.5) containing 0.1% Triton X-100 and sonicated at 100 W for 10 min with an Insonator model 201M (Kubota).Next, trichloroacetic acid was added (final concentration 10%), and the samples were vortexed and incubated at 4°C for 1 h. Then, the leaves were removed, and the samples were centrifuged. Subsequently, the pellet was washed twice with acetone and resuspended in a buffer containing 50 mM Tris-HCl (pH 7.5). The samples were denatured by boiling in SDS sample buffer and then subjected to SDS-PAGE analysis. The lipidated and non-lipidated forms of Atg8 were separated using SDS-PAGE containing 6 M Urea [28].

## Supporting Information

Figure S1 **Cell-based methanol assay on growing** ***A. thaliana*** **leaves (2–3 weeks after germination).** Leaves were inoculated with the *C. boidinii* PDAS strain at 12 hh, when the estimated methanol concentration on the leaves was lowest during the daily light–dark cycle. The cell suspension inoculated on the plant leaves contained 0, 2.5, or 250 mM methanol. The fluorescent intensity was measured 4 h after inoculation. The methanol concentration represents the average from at least 10 cells. Error bars show the standard deviations of measured cells.
(EPS)

Figure S2 ***Pichia pastoris*** **proliferation on growing** ***A. thaliana*** **leaves observed under a fluorescent microscope.** Confocal microscope images of the YFP-labeled wild-type strain on a plant leaf. *P. pastoris* cells were spotted on *Arabidopsis* leaves (2–3 weeks after germination). Bar, 10 µm.
(EPS)

Figure S3 **Peroxisomes were not observed in** ***C. boidinii pex5*** **Δ strain expressing Venus-PTS1 under the control of the** ***ACT1*** **promoter.** *C. boidinii* cells inoculated on (A) a growing plant leaf, (B) wilting plant leaf observed at the indicated time. Bar, 5 µm.
(EPS)

Figure S4 ***C. boidinii*** **did not proliferate on leaves of dead** ***A. thaliana*** **.** Quantitation of the cell mass of the wild-type and *aod1*Δ strains after 1, 4, 7, and 11 days. Error bars show the standard deviations of triplicate experiments.
(EPS)

Figure S5 **Disruption of** ***ATG*** **genes in** ***C. boidinii*** **.** Southern analyses were conducted for *Pvu*II-, *Pst*I-, and *Ava*II-digested DNA extracted from *ATG1-, ATG8-*, and *ATG30*-deleted *C. boidinii* strains together with the wild-type strain using a 0.7-kb *Pst*I-*Bam*HI fragment from the upstream region of the *CbATG1* gene, a 0.7-kb *Pst*I-*Cla*I fragment from the upstream region of the *CbATG8* gene, and a 0.6-kb *Bgl*II-*Ava*II fragment from the downstream region of the *CbATG30* gene as the hybridization probes, respectively.
(EPS)

Figure S6 **Processing of Venus-Atg8 was inhibited in** ***atg1Δ*** **, but not in** ***atg30Δ*** **strain, under nitrogen starvation conditions.** Cells grown on YPD medium were shifted to synthetic dextrose medium without a nitrogen source, and the cell extracts were subjected to Western analysis using an anti-GFP polyclonal antibody.
(EPS)

Figure S7 **Impaired pexophagy in the** ***atg1Δ*** **,** ***atg8Δ*** **, and** ***atg30Δ*** **strain.** Cells grown on YPM medium were shifted to synthetic ethanol medium, and the cell extracts were subjected to Western analysis using an anti-AOD polyclonal antibody or an anti-β-actin monoclonal antibody.
(EPS)

Table S1 **List of oligonucleotide primers.**
(DOC)

Text S1 **Materials and Methods (Supplementary).**
(DOC)
