# Supplementary material for: Yeast Methylotrophy and Autophagy in a Methanol-Oscillating Environment on Growing Arabidopsis thaliana Leaves
Source: PLoS One. 2011 Sep 26;6(9):e25257. doi: 10.1371/journal.pone.0025257 (PMC3180373; doi:10.1371/journal.pone.0025257)
Supplement: Table S1 — List of oligonucleotide primers. (DOC) [file pone.0025257.s008.doc]

**Table S1 List of oligonucleotide primers**

Primer name 5’- sequence-3’

Fw-Zcb GGCCACACACCATAGCTTCAAAATGTTTCTA

Rv-Zcb GGCCCAGCTTGCAAATTAAAGCCTTCGA

Fw-Pcta-EcoRI GGAATTCACTAGTCTTAAGGTTTCCATAGC

Fw-Pdas-EcoRI GGAATTCAAAATGTGGAGAAAAGAAATC

Fw-Pfdh GTCAACAAATCAATCAGCCAATCTACCAAT

Fw-Pfgh-EcoRI GGAATTCGTTTGATATCTGTTATTTAAATG

Fw-Pfld-EcoRI GGAATTCTCTAAGTTTTAATCTTACAAACA

Rv-Pcta-Not ATAAGAATGCGGCCGCGGTACCATTTGAATATAATTTATT

Rv-Pdas-Not ATAAGAATGCGGCCGCTTTTGTAATTTTTTTTAATAATT

Rv-Pfdh-Not ATAAGAATGCGGCCGCGGTACCTTTGTAAAGTCAATAAT ATAATATATAATC

Rv-Pfgh-Not ATAAGAATGCGGCCGCGGTACCTTTTTTAATTATTATAAT

Rv-Pfld-Not ATAAGAATGCGGCCGCGGTACCTTTTGTGGAATAAAAAATAGAT AAATATGA

Fw-Qvenus CAGTTCCATGGCCAACTTT

Rv-Qvenus CTTCTGGCATAGCAGATTTGAAG

Fw-cbact1-RT GAAGCCCAATCCAAGAGAGG

Fw-cbaod1-RT TTCGATGCATTTGTCTCTGG

Fw-cbdas1-RT AGGTGGTCATCCTGGTTCTG

Fw-cbfld1-RT CCAGAAGCTCCAAAGGATAG

Fw-cbfgh1-RT GATTTCGGTACAGGTGCTGG

Fw-cbcta1-RT TAACTGCCCAATGAGAGCTG

Fw-cbpmp20-RT CTTCCCAACCACTGACGATG

Rv-cbact1-RT GTGTTCTTCTGGGGCAACTC

Rv-cbaod1-RT TGCCAATTCAGCTTCTGTTG

Rv-cbdas1-RT TCACGATTGAACCATGTTGG

Rv-cbfld1-RT GCAACAGCACCTTGAATAAC

Rv-cbfgh1-RT ACCACCCATTGAATGACCTG

Rv-cbcta1-RT GATATTGAACCGGGCAGTTG

Rv-cbpmp20-RT GTGTGAATGCACCTGGAACTG

Fw-cbATG1-5EcoRI GGATCCCACCATTTGAATCACATAATCACG

Rv-cbATG1-5Not GCGGCCGCTGTGTATGTGTATATAAG

Fw-cbATG1-3Xho CTCGAGCCCTTTGTGTTTGTTGAGTTAC

Rv-cbATG1-3EcoRI GGATCCCCCCAATACATACAATAGCAAC

Fw-PcbATG8-Xho CCGCTCGAGGTCTGTATCTATCTCTGTC

Rv-PcbATG8-Kpn GGGGTACCTTTTGTGACTGTGTTTATATAA

Fw-cbATG8-Kpn GGGGTACCATGAAATCACAATTTAAAAGCG

Rv-cbATG8-Pst AACTGCAGGAAAGAGAGGATATGGATAAAG

Fw-HA-cbATG8 TTCCAGATTACGCTGGTTATCCATATGATGTTCCAGATTACGC TATGAAATCACAATTTAAAAGCGAACA

Rv-HA-cbATG8 CATCATATGGATAACCAGCGTAATCTGGAACATCATATGGATA TTTTGTGACTGTGTTTATATAAGATTA

Fw-cbATG8-5Bam GGATCCCGTTAAGGAATAAGAGAAGC

Rv-cbATG8-5Not GCGGCCGCCGATAAGATTGAAGATG

Fw-cbATG8-3Xho CTCGAGCCATATCCTCTCTTTCTTTTC

Rv-cbATG8-3Bam GGATCCGCAGTTACACATGCTATAAG

Fw-cbATG30-5EcoRI GGAATTCGCGTATTTTATTGACAGAATGG

Rv-cbATG30-5Bam CGGGATCCGTGTTTTGAATGCGGTCC

Fw-cbATG30-3Cla CCATCGATGCTGGTAATAAAGACAGCAAAG

Rv-cbATG30-3EcoRI GGAATTCGTATCCATTAACAACCGGTAATC
