# Supplementary material for: Yeast Methylotrophy and Autophagy in a Methanol-Oscillating Environment on Growing Arabidopsis thaliana Leaves
Source: PLoS One. 2011 Sep 26;6(9):e25257. doi: 10.1371/journal.pone.0025257 (PMC3180373; doi:10.1371/journal.pone.0025257)
Supplement: Text S1 — Materials and Methods (Supplementary). (DOC) [file pone.0025257.s009.doc]

**Materials and Methods (Supplementary)**

**Media and culture conditions**

The yeast strains were grown in YPD (1% yeast extract, 2% peptone, and 2% glucose), SD (0.67% yeast nitrogen base without amino acids and 2% glucose, supplemented with an appropriate amino acid) or SM (0.67% yeast nitrogen base without amino acids and 0.7% methanol) medium. The initial pH of the medium was adjusted to 6.0. The yeast were grown at 28°C under aerobic conditions with reciprocal shaking, and growth was monitored by measuring the optical density at 600 nm (OD_600_).

**Construction of the Venus expression plasmids**

The backbone plasmids with different promoter sequences for the *C. boidinii* genes were prepared as follows: the 5’ untranslated regions of *CTA1*, *DAS1*, *FGH1*, and *FLD1* were PCR amplified with primer sets Fw-Pcta-EcoRI/Rv-Pcta-Not, Fw-Pdas-EcoRI/Rv-Pdas-Not, Fw-Pfgh-EcoRI/Rv-Pfgh-Not and Fw-Pfld-EcoRI/ Rv-Pfld-Not, respectively, excised with *Eco*RI/*Not*I, and ligated with the *Eco*RI/*Not*I fragment of pNOTe1 [1] containing the *AOD1* terminator and *URA3* regions. The promoter region of *FDH1* was PCR amplified with primer set Fw-Pfdh/Rv-Pfdh-Not, and ligated with the pNOTe1 fragment whose ends were blunted with KOD DNA polymerase (TOYOBO).

To express Venus (a modified form of yellow fluorescent protein) or its derivative protein with peroxisome targeting signal 1 (PTS1) under various promoters in *C. boidinii*, codon usage in the Venus coding sequence was optimized based on other protein coding sequences in this organism. DNA fragments encoding Venus or Venus-PTS1 with optimized codons were cloned into pACT1 [2] using the *Sal*I and *Pst*I sites at the 5’ or 3’ ends of the fluorescent protein coding sequences, respectively, to yield pACT1V (Venus under the *ACT1* promoter) and pACTVPTS (Venus-PTS1 under the *ACT1* promoter). Similarly, *Not*I restriction sites were used as linker sites, and the PCR-amplified fragments encoding Venus or Venus-PTS1 were introduced into pNOTe1 or the backbone plasmid harboring the *DAS1* promoter region. This yielded pAODV (Venus under the *AOD1* promoter), pDASV (Venus under the *DAS1* promoter), and pDASPTS (Venus-PTS1 under the *DAS1* promoter). *Kpn*I and *Not*I restriction sites were used as linker sites to generate pCTAV (Venus under the *CTA1* promoter), pFDHV (Venus under the *FDH1* promoter), pFGHV (Venus under the *FGH1* promoter), and pFLDV (Venus under the *FLD1* promoter). The DNA fragment encoding Venus was ligated into the *Sal*I-*Pst*I sites of p20PU [3] to yield pPMP20V (Venus under the *PMP20* promoter).

The nucleotide sequences of *VENUS* (720 bp) will appear in the DDBJ/EMBL/GenBank nucleotide sequence databases with the accession number AB634497.

**Disruption of *ATG* genes in *C. boidinii***

A 1.2-kb fragment encoding the Zeocin resistance gene with codon usage optimized for *C. boidinii* was PCR amplified using primers Fw-Zcb and Rv-Zcb with pREMI-Zc [4] as the template. This fragment was ligated into the *Nae*I site of SK^+^ to generate SK^+^-Zc.

A 1.4-kb region upstream of the *CbATG1* gene was PCR amplified with primers Fw-cbATG1-5EcoRI and Rv-cbATG1-5Not using genomic DNA as the template. Similarly, the 1.0-kb region downstream of the *CbATG1* gene was PCR amplified with primers Fw-cbATG1-3Xho and Rv-cbATG1-3EcoRI. These PCR products were ligated into the *Eco*RV site of SK^+^ and then digested with *Eco*RI and *Not*I or *Eco*RI and *Xho*I. These fragments were recombined with the 4.2-kb *Xho*I-*Not*I fragment of the SK^+^-Zc plasmid, yielding the *CbATG1* disruption vector pDatg1. After linearizing with *Eco*RI, pDatg1 was transformed into the wild-type strain using the modified lithium acetate method. Zeocin-resistant colonies were selected on YPD medium supplemented with Zeocin. Disruption of the *CbATG1* gene was confirmed by Southern blot analysis with *Pvu*II-digested genomic DNA of the transformant and a 0.7-kb *Pst*I-*Bam*HI fragment from the upstream region of the *CbATG1* gene as the probes.

The isogenic *Cbatg8*∆ and *Cbatg30*∆ mutant strains were generated by replacing the respective gene coding regions with the fragment of the SK^+^-Zc plasmid as described above.

The nucleotide sequences of *CbATG1* (657 bp), *CbATG8* (500 bp) and *CbATG30* (1806 bp) will appear in the DDBJ/EMBL/GenBank nucleotide sequence databases with the accession numbers, AB634494, AB634495 and AB634496, respectively.

**Fluorescence** **microscopy and image analysis**

Yeast cells on the plant surface were observed under a Zeiss LSM510 META laser scanning confocal microscope equipped with a Plan-FLUAR 100x, NA 1.45 oil objective on an inverted microscope Axiovert 200M (Zeiss). Venus was excited with a multiline (458, 477, 488, and 514 nm) argon laser with a 530−600 nm filter for emission. An HFT 405/514 beam splitter was used as a connecting filter.

The *C. boidinii* PDAS strain expressing Venus-PTS1 under the control of the *DAS1* promoter grown on SD medium was washed twice and resuspended in sterilized water. Then, this suspension was spotted onto YLN (0.17% yeast nitrogen base without amino acids and 0.05% ammonium sulfate) agar plates containing various concentrations of methanol or onto *A. thaliana* leaves*.* After 4 h incubation, the cells were resuspended in sterilized water and observed with a Zeiss LSM510 META laser scanning confocal microscope. Four or five fields containing at least 10 individual cells were captured per sample. The samples were observed under the same conditions with regard to the laser output (50%), pinhole Φ (3.00 Airy Unit) and PMT gain setting (600). The intensity of the fluorescent images were determined with a Carl Zeiss LSM Image Examiner “Histogram” program. The mean pixel intensity per cell was computed based on the sum of the intensities of all of the individual pixels averaged over the total number of pixels covering the cells. The threshold for the pixel intensity was set at 100 through 255. The methanol concentration was estimated from the average fluorescence collected from at least 50 cells.

**Quantitative PCR**

To quantitate yeast cell proliferation at phyllosphere, qPCR was performed in a 20-µl mixture in glass capillary tubes using a LightCycler (Roche Diagnostic). The PCR cycling reaction for the sample DNA was performed with 1x SYBR *Premix Ex Taq* (TaKaRa) according to the following parameters: first cycle, 30 s of denaturation at 95°C; second cycle with 40 repetitions, 95°C for 5 s, 60°C for 20 s (all temperature transitions, 20°C s^−1^). The primers Fw-Qvenus and Rv-Qvenus were used for the reactions, and pACTV was used to generate a standard curve.

To follow the yeast gene expression at phyllosphere, total RNA was extracted from *C. boidinii* on *A. thaliana* using ISOGEN (Nippon Gene). cDNA was synthesized from 2 µg of total RNA using Random Primers (Promega) and ReverTra Ace (Toyobo). After the RNA was reverse transcribed for 50 min at 42°C, the samples were heated for 5 min at 99°C to terminate the reaction, and 0.5 µl of RNase H was added. qRT-PCR was performed in a 20-µl mixture in glass capillary tubes using a LightCycler (Roche Diagnostic) as described above. The relative abundance of mRNA was standardized based on the *CbACT1* levels.

***CbATG8* expression in *C. boidinii***

A strain expressing a Venus-tagged Atg8 (Venus-Atg8) was constructed as follows. First, a fragment with the 0.7-kb 5’ untranslated region and 0.5-kb *CbATG*8 coding region was generated by PCR using primers Fw-PcbATG8-Xho and Rv-cbATG8-Pst and genomic DNA as the template. The PCR product was digested with *Xho*I and *Pst*I , and ligated with the 6.0-kb *Xho*I-*Pst*I fragment of pACT1 to form pEX-ATG8. Next, a 7.5-kb fragment was amplified by inverse PCR using the primer set Fw-cbATG8-Kpn/ Rv-PcbATG8-Kpn and pEX-ATG8 as the template. The Venus-coding region was PCR amplified with primer set Fw-venus-Kpn / Rv-venus-end-Kpn. These two fragments were digested with *Kpn*I and ligated to form pEX-Venus-ATG8. The resulting plasmid was linearized with *Eco*RV and then introduced into the wild-type strain and *Cbatg1*∆ mutant strain. Next, a strain expressing HA-tagged Atg8 (HA-Atg8) was constructed as follows. The 7.6-kb fragment was amplified by inverse PCR using primer set Fw-HA-cbATG8/Rv-HA-cbATG8 and pEX-ATG8 as the template, and then this fragment was self-ligated to form pEX-HA-ATG8. The resulting plasmid was linearized with *Eco*RV and introduced into the wild-type strain.

**Immunoblot analysis**

After electrotransferring the proteins to membranes, the blot was blocked for more than 1 h in 5% skim milk in TBS-T buffer (20 mM Tris-HCl, 137 mM NaCl, and 0.1% Tween-20). The blots were incubated with an anti-GFP antibody (1:1000 dilution) , an anti-HA antibody (1:1000 dilution), an anti-AOD antibody (1:10000 dilution), or an anti-β-actin antibody (1:1000 dilution) in TBS-T buffer for 1 h with gentle shaking, washed three times in TBS-T, and incubated with an anti-mouse IgG HRP-conjugated antibody (1:5000 dilution) in TBS-T buffer for 1 h. The blots were washed three times in TBS-T, and immunoreactive bands were detected using Western Lighting Chemiluminescence Reagent Plus (Perkin-Elmer Life Sciences).

**References (Supplementary)**

1. Sakai Y, Akiyama M, Kondoh H, Shibano Y, Kato N (1996) High-level secretion of fungal glucoamylase using the *Candida* *boidinii* gene expression system. Biochim Biophys Acta 1308: 81-87.

2. Sakai Y, Yurimoto H, Matsuo H, Kato N (1998) Regulation of peroxisomal proteins and organelle proliferation by multiple carbon sources in the methylotrophic yeast, *Candida* *boidinii*. Yeast 14: 1175-1187.

3. Yurimoto H, Komeda T, Lim CR, Nakagawa T, Kondo K, et al. (2000) Regulation and evaluation of five methanol-inducible promoters in the methylotrophic yeast *Candida* *boidinii*. Biochim Biophys Acta 1493: 56-63.

4. Sasano Y, Yurimoto H, Sakai Y (2007) Gene-tagging mutagenesis in the methylotrophic yeast *Candida* *boidinii*. J Biosci Bioeng 104: 86-89.
